# Supplementary material for: Complexome profiling of the Chlamydomonas psb28 mutant reveals TEF5 as an early PSII assembly factor
Source: Plant Cell. 2025 Mar 17;37(6):koaf055. doi: 10.1093/plcell/koaf055 (PMC12164590; doi:10.1093/plcell/koaf055)
Supplement: koaf055_Supplementary_Data [file koaf055_supplementary_data.zip › psb28_interactive.zip!/plots/intensity/qualityPlots/peptides/ATP2_Cre17.g698000.t1.2.html]

### Cre17.g698000.t1.2

Trivial name: ATP2  
SeqCoverage [median]: 64.30  
Mass: 61.82 kDa  
  
 

| Sequence | MissedCleavage | Mass [Da] | wtIntensity | muIntensity | Score |
| --- | --- | --- | --- | --- | --- |
| ADKLAKDVAAR | 2 | 1156.66 | 0.00 | 784.77 | 83.14 |
| AEAISSENMVLNEK | 0 | 1533.73 | 0.00 | 1104.01 | 107.85 |
| AHGGFSVFAGVGER | 0 | 1389.68 | 161020.02 | 417130.89 | 139.78 |
| AKSSEALKDVPSLEK | 2 | 1600.87 | 11695.18 | 3840.01 | 98.94 |
| DTISAFTGILQGK | 0 | 1349.72 | 28304.60 | 17335.24 | 243.53 |
| EAPEFTEQSTEQEILVTGIK | 0 | 2248.11 | 119128.52 | 196248.66 | 217.73 |
| EGNDLYR | 0 | 865.39 | 27206.72 | 42707.78 | 131.04 |
| EGNDLYREMIESGVIK | 1 | 1851.90 | 115247.12 | 172900.34 | 161.08 |
| EMIESGVIK | 0 | 1004.52 | 99211.43 | 121610.66 | 175.38 |
| FDGELPSILSALEVQGHNVR | 0 | 2180.12 | 16391.09 | 121870.65 | 146.10 |
| FTQANSEVSALLGR | 0 | 1491.77 | 400293.88 | 757109.79 | 333.58 |
| GEKVPLPK | 1 | 866.52 | 15752.94 | 21297.60 | 118.74 |
| GQKVVNTGSPIKVPVGR | 2 | 1735.01 | 0.00 | 2655.84 | 107.74 |
| GSITSVQAVYVPADDLTDPAPATTFAHLDATTVLSR | 0 | 3699.86 | 1402.69 | 0.00 | 36.93 |
| IPSAVGYQPTLATDLGGLQER | 0 | 2185.14 | 315742.68 | 367038.38 | 199.37 |
| LVLEVAQHMGDNTVR | 0 | 1680.86 | 335675.27 | 459536.29 | 176.91 |
| MAGEIKDEVIDADDSLEEDFK | 1 | 2368.06 | 0.00 | 2331.36 | 70.45 |
| MLNPNIIGAEHYNIAR | 0 | 1824.93 | 337928.49 | 1154480.15 | 191.78 |
| SIAELGIYPAVDPLDSTSR | 0 | 2003.02 | 264940.36 | 373220.96 | 259.48 |
| SSEALKDVPSLEK | 1 | 1401.74 | 435615.12 | 492006.51 | 237.21 |
| TREGNDLYR | 1 | 1122.54 | 31014.08 | 37786.28 | 146.11 |
| TREGNDLYREMIESGVIK | 2 | 2109.05 | 34853.34 | 57448.45 | 137.44 |
| TVLIMELINNVAK | 0 | 1456.83 | 134514.98 | 177672.99 | 330.41 |
| VALTGLTVAEYFR | 0 | 1438.78 | 70801.74 | 136505.72 | 268.73 |
| VLQDYK | 0 | 764.41 | 200027.66 | 380579.57 | 153.67 |
| VVDLLAPYQR | 0 | 1172.66 | 689198.31 | 1593745.78 | 180.93 |
| VVNTGSPIK | 0 | 913.52 | 78734.34 | 140078.36 | 168.27 |
| VVNTGSPIKVPVGR | 1 | 1421.84 | 580872.11 | 755109.22 | 210.91 |
| YDDLPEMAFYMVGGIHEVVEK | 0 | 2441.13 | 44709.97 | 148248.78 | 132.67 |
|  |  |  | 4550282.6 | 8152385.0 |  |
